# JBrowseR: an R interface to the JBrowse 2 genome browser

**DOI:** 10.1093/bioinformatics/btab459

**Published:** 2021-07-01

**Authors:** Elliot A Hershberg, Garrett Stevens, Colin Diesh, Peter Xie, Teresa De Jesus Martinez, Robert Buels, Lincoln Stein, Ian Holmes

**Affiliations:** Department of Bioengineering, University of California, Berkeley, Berkeley, CA 94720, USA; Department of Bioengineering, University of California, Berkeley, Berkeley, CA 94720, USA; Department of Bioengineering, University of California, Berkeley, Berkeley, CA 94720, USA; Department of Bioengineering, University of California, Berkeley, Berkeley, CA 94720, USA; Department of Bioengineering, University of California, Berkeley, Berkeley, CA 94720, USA; Department of Bioengineering, University of California, Berkeley, Berkeley, CA 94720, USA; Adaptive Oncology, Ontario Institute for Cancer Research, Toronto, ON M5G 0A3, Canada; Department of Bioengineering, University of California, Berkeley, Berkeley, CA 94720, USA

## Abstract

**Motivation:**

Genome browsers are an essential tool in genome analysis. Modern genome browsers enable complex and interactive visualization of a wide variety of genomic data modalities. While such browsers are very powerful, they can be challenging to configure and program for bioinformaticians lacking expertise in web development.

**Results:**

We have developed an R package that provides an interface to the JBrowse 2 genome browser. The package can be used to configure and customize the browser entirely with R code. The browser can be deployed from the R console, or embedded in Shiny applications or R Markdown documents.

**Availability and implementation:**

JBrowseR is available for download from CRAN, and the source code is openly available from the Github repository at https://github.com/GMOD/JBrowseR/.

## 1 Introduction

The development of genome browsers has been described as a milestone of the genomic revolution (Packer, 2007). Genome browsers provide researchers with the ability to visualize and explore genomic annotations and data. Due to their widespread adoption and use, the linear display of genomic information along reference coordinates is one of the most common representations of biological data in the 21st century.

Since their original development during the advent of genome sequencing (Birney *et al.*, 2004; Kent *et al.*, 2002), genome browsers have made considerable gains in performance and sophistication. One important development has been the implementation of genome browsers in JavaScript, beginning with JBrowse (Buels *et al.*, 2016). Leveraging JavaScript—along with modern web technologies such as Canvas and SVG—makes it possible to move computation that previously took place on a server into the client web browser, as well as offering a more responsive and interactive experience to the user.

We have recently released JBrowse 2, which is an extensible platform for visualizing and integrating biological data, consisting of a ground-up rewrite of JBrowse using ReactJS and TypeScript. JBrowse 2 contains many new views and components, to be described in a later paper. However, one core part is a React component that renders a configurable linear genome browser, enabling researchers to embed custom browsers into existing React applications. While the JBrowse 2 React linear genome view component is powerful and extensible, its use can present an obstacle to bioinformaticians who do not have experience with React development.

In contrast to React and JavaScript, the R programming language and environment are widely used in the bioinformatics community. R also has a strong history of tools for interacting with genome browsers, such as the rtracklayer (Lawrence *et al.*, 2009) package for interacting with the UCSC genome browser, igvR for accessing igv.js (Robinson *et al.*, 2011, 2017), and epivizr for manipulating epiviz (Chelaru *et al.*, 2014).

Although these tools exist for interacting with genome browsers from R, they all work by providing a communication channel between an app in the browser and an R environment. It is not possible to use these packages to render visualizations in popular new tools such as R Shiny apps or R Markdown documents. In order to provide a novel tool and make JBrowse 2 accessible to the R community, we introduce JBrowseR: an R interface the JBrowse 2 genome browser. JBrowseR is an R package with functions for embedding a custom browser instance in a Shiny app, R Markdown document, or the R console.

## 2 Materials and methods

JBrowseR is implemented as an R package and distributed on CRAN. The package was built according to the R best practices encoded in the devtools package, ensuring continual use of R CMD Check and maintaining consistent and robust function documentation during development. The core rendering methods of the library rely on the htmlwidgets framework, which can be used to embed JavaScript visualization tools in R Shiny apps, as well as R Markdown documents. Htmlwidgets can also be used from an R interactive console. Using the reactR package, JBrowseR renders the JBrowse 2 widget inside of a root HTML element in an htmlwidget.

The interface of JBrowseR enables users to generate JBrowse 2 configuration for their own data using simple R functions. The configuration values can be composed together to create an arbitrarily complex custom browser. The majority of genomic data formats commonly displayed in genome browsers are supported, including (but not limited to) FASTA, BED, GFF3, BAM, CRAM, VCF, Wiggle and bigWig. JBrowseR also includes an HTTP server for serving local data that is configured with the necessary settings and features for working with genome browsers such as JBrowse and IGV.js (Robinson *et al.*, 2011, 2017).

In addition to loading data from bioinformatics files, users can create tracks from R data frames. This provides an interface for users to generate computational results in R and pass them directly to the browser. When used in Shiny, JBrowseR can also send messages back to the app, enabling powerful integration with downstream processes. We provide an example app using this feature to create a data frame of bookmarked features from the browser.

The source code for JBrowseR is hosted on GitHub and is automatically tested using continuous integration running on the Windows, MacOS and Linux operating systems. Automated tests for the package are implemented using the testthat R package.

## 3 Results and discussion

**Fig. 1.**
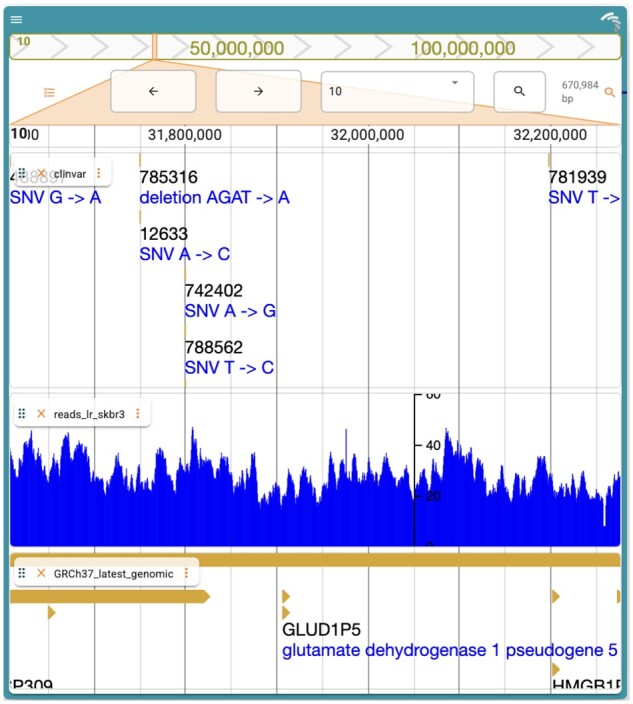
A custom JBrowse 2 genome browser generated using JBrowseR.

In order to demonstrate the utility of JBrowseR, several Shiny apps were built and are included along with the package source code on GitHub. One of the included apps demonstrates adding CRAM, VCF, GFF3, and bigWig data (Fig. 1), as well as setting a custom color palette for the browser. Another one of the provided demo apps illustrates how JBrowseR can also be configured with a JSON file (like other JBrowse 2 apps) by loading the SARS-CoV-2 reference genome and NCBI annotations from a JBrowse 2 configuration file. Finally, we have provided an example deployment of JBrowseR in an app with interactions connected to other R shiny UI components at https://elliothershberg.shinyapps.io/sars-cov-2-spike-mutations/.

One of the core strengths of JBrowseR is its versatility. It considerably lowers the level of expertise required to create a web-facing genome database with a fast and flexible genome annotation browser. However, web applications are not the only available target point for the rendering functions: JBrowseR can also embed a genome browser into R Markdown, a flexible documentation format that is used to write scientific articles (including this one). We anticipate that as platforms such as eLife’s ‘reproducible article’ (Maciocci *et al.*, 2019) mature and become more widely adopted, it will be possible for genomics articles to contain interactive genome browsers such as JBrowseR displaying their data.

## 4 Availability

JBrowseR is freely available for download from CRAN, and the source code is publicly available at https://github.com/GMOD/JBrowseR/. The package reference guide and tutorials can be found at https://gmod.github.io/JBrowseR/.
